# Evaluating the Real‐World Implementation and Effectiveness of a Collaborative Care Model for Adults with Depression and Anxiety at an Urban, Academic Hospital

**DOI:** 10.1111/jep.70332

**Published:** 2025-12-01

**Authors:** Peter J. Hahn, Daniel Y. Johnson, Joanne Wang, Carly Lusk, Samantha Allen, Fabiana S. Araújo, Daniel Yohanna, Erin M. Staab, Neda Laiteerapong

**Affiliations:** ^1^ Pritzker School of Medicine University of Chicago Chicago Illinois USA; ^2^ Section of General Internal Medicine, Department of Medicine University of Chicago Chicago Illinois USA; ^3^ University of Chicago Medical Center Chicago Illinois USA; ^4^ Department of Psychiatry and Behavioral Neuroscience University of Chicago Chicago Illinois USA

## Abstract

**Rationale:**

Over 30% of adults in the United States have symptoms of depressive or anxiety disorders. The majority of these patients are treated only in primary care and have suboptimal outcomes. The Collaborative Care Model (CoCM) is designed to address this problem.

**Aims and Objectives:**

This study evaluated the implementation and effectiveness of CoCM at an urban academic medical center primary care clinic.

**Methods:**

Retrospective chart review and analysis informed by the RE‐AIM framework.

**Results:**

Across 148 patients, mean PHQ‐9 scores decreased by 4.8 (SD, 5.8, *p* < 0.001) and GAD‐7 scores decreased by 5.6 (SD, 5.7, *p* < 0.001). Overall adoption rate was 36% and differed between attending physicians/APNs (50%) and residents (32%). Implementation showed fidelity to measurement‐based care, but only 40% of cases were reviewed with the psychiatrist.

**Conclusion:**

A real‐world CoCM can improve symptoms of depression and anxiety for adults; however, trainee adoption and adherence to psychiatric case reviews may merit special attention.

## 1 Introduction

Primary care clinicians (PCCs) are the “de facto” mental health care providers in the United States and care for over half of patients with depression and anxiety [1]. Up to 90% of patients with depression and anxiety are treated in primary care [2], however, only about half of patients in primary care are diagnosed, only half of those who are diagnosed receive any treatment, and, finally, only half of those who receive any treatment receive adequate treatment [3].

One solution to address inadequate treatment of mental health conditions in primary care has been the Collaborative Care Model (CoCM) [4]. The CoCM integrates behavioral health care with primary care and emphasizes (1) a multi‐professional approach to patient care, (2) a structured management plan, (3) scheduled patient follow‐ups, and (4) enhanced inter‐professional communication [5]. Previous randomized trials have found that CoCM generally leads to significant improvements for patients with depression and anxiety [6]. However, few real‐world studies describe the process of implementation or quantify the effectiveness of CoCM in the real world as opposed to clinical trials [7].

Recognizing the heterogeneity in real‐world effectiveness, it is important to examine the implementation of CoCM programs. The CoCM program at UChicago Medicine faces unique challenges because of its location in the south side of Chicago, a primary care and mental health shortage area. In addition, the clinic is a teaching environment with both attendings and residents that staff the clinic part‐time. The lessons that can be drawn from this particular implementation of the model can inform other efforts to establish the CoCM at academic institutions as well as in primary care settings.

## 2 Methods

### 2.1 Design

The goal of this study was to evaluate the first‐year implementation of the CoCM at UChicago Medicine. To accomplish this, a team of researchers performed a retrospective chart review for adult patients with depression and/or anxiety that enrolled in the CoCM program from inception in September 2021 to September 2022. This study was determined to be a Quality Improvement project by the University of Chicago Biological Sciences Division and was not reviewed by the Institutional Review Board. In addition, the authors declare that there is no conflict of interest and received no financial support for the research, authorship, and/or publication of this article.

#### 2.1.1 CoCM Program Setting and Description

The CoCM program was initiated within the academic faculty and the teaching general internal medicine clinic at UChicago Medicine in September 2021. This clinic is responsible for providing primary care to about 30,000 patients with an insurance mix of 47% private insurance, 43% Medicare, and 10% dual Medicare‐Medicaid. Patient demographics are as follows: 58% Black, 28% White, 5% Asian, 5% Hispanic, and 4% Other/Unknown. < 4% of patients are under 18 years old, 26% are 19−49, 23% are 50−64, 31% are 65−79, and 17% are 80 years or older. 67% of clinic patients are female. There were 37 attending physicians, 3 advanced practice nurses, and 117 resident physicians. Other personnel included 3 registered nurses, 2 licensed practical nurses, 15 medical assistants, and 1 general medical social worker.

The CoCM at UChicago Medicine was designed using the foundation of the University of Washington AIMS Center [8]. The program served three distinct populations: adults 18 years or older with depression and/or anxiety, adolescents 12–17 years old with depression and/or anxiety, and adults 50 years or older with cognitive impairment and neuropsychiatric symptoms. This evaluation focused on the adult depression/anxiety CoCM, which included 1 medical director (N.L.), 1 consulting psychiatrist (D.Y.), 1 consulting psychologist (F.A.), and two licensed clinical social workers (S.A. and C.L.).

For patients to be eligible for the adult depression/anxiety CoCM, they needed to meet the following inclusion criteria: (1) receiving primary care at the clinic with an appointment in the last year, (2) at least 18 years of age, (3) moderate or severe depression and/or anxiety symptoms, that is, PHQ‐9 and/or GAD‐7 ≥ 10, and (4) no other active mental health condition, frequent passive suicidality, or actively being managed by a psychiatrist. The PHQ‐9 questionnaire measures the frequency of nine depression symptoms over the past 2 weeks [9]. The GAD‐7 questionnaire measures the frequency of seven symptoms related to generalized anxiety disorder [10].

PCCs were trained on the CoCM program through a series of group information sessions and via email. During the first 6 months of the program, the program director (N.L.) also reviewed cases of (1) patients who had PHQ‐9 or GAD‐7 score ≥ 10 or (2) patients referred to other behavioral health services for depression or anxiety, and if patients met eligibility criteria, sent emails to PCCs reminding them about the CoCM program and how to refer their patients.

Referred patients were connected with a social worker who (1) conducted a diagnostic assessment and confirmed eligibility, (2) provided care management, (3) consulted with a psychiatrist for patients for whom they had diagnostic uncertainty or for medication questions (i.e., case review), and (4) measured symptoms using the PHQ‐9 and/or GAD‐7 at least monthly. Additionally, this CoCM program emphasized the provision of brief therapeutic interventions. According to program guidelines, a patient could remain enrolled in this program for 3 months. Social workers met with the consulting psychiatrist weekly for 30 min to review new cases and cases in which patients were not clinically improving. Social workers also met with the consulting psychologist for 1 h per week to debrief on clinical cases.

Patients could complete the program in multiple ways. Graduation was defined by 50% reduction in PHQ‐9 and/or GAD‐7 scores sustained for 1 month. Patients could also complete CoCM if they were referred to long‐term therapy and had successfully attended at least 2 appointments, or if they were referred to a psychiatrist and had attended at least one appointment. Patients were referred to psychiatry if their depression or anxiety symptoms were identified to be related to another primary mental health condition. If patients missed two appointments in a row without explanation, they were discharged from the program at the social worker's discretion.

### 2.2 Data Sources

There were two data sources for this study: (1) the CoCM registry, which included demographic information on enrolled patients and (2) the electronic health record, which contained documentation on each clinical encounter and case review. Patient charts were reviewed by two independent coders.

### 2.3 Evaluation

This evaluation was informed by the RE‐AIM planning and evaluation framework (Table 1) [11]. Reach was defined as the number and characteristics of individuals that enrolled in the CoCM program. Effectiveness was assessed by changes in PHQ‐9 and GAD‐7 scores from referral to final assessment (i.e., graduation or end of the study period). Adoption reflected provider uptake and was measured by the proportion of PCCs who referred to the program. Implementation was characterized by several factors related to intervention delivery and fidelity, including the frequency of encounters between the patient and the social worker, proportion of patients that were referred to and started long‐term therapy, proportion of patients discussed during case review, case review outcomes, frequency of assessments, and time enrolled in the program.

**Table 1:** Evaluation of collaborative care program based on RE‐AIM framework.

	Definition	Strategies	Measures
Reach	How many patients within the target population participate in the intervention	Posting fliers in the clinic; using EHR reports to identify more eligible patients	Number and characteristics of patients enrolled
Effectiveness	Impact of the intervention on important outcomes	Measurement‐based care; regular meeting cadence for psychiatric case review and psychologist supervision; brief therapeutic interventions	Change in depression and anxiety scores; % of patients achieving response
Adoption	How many and which types of providers use the program	Training for providers; reminders and personal outreach	% of providers referring overall and by attending versus resident
Implementation	Extent to which the program is delivered as intended	Onboarding for social workers; regular meetings between social workers and program director; documentation in EHR and registry	Rate of encounters, case reviews, and symptom assessments
Maintenance	Extent to which the program is sustained over time	Established billing procedures; leadership support	*Not assessed due to short timeframe of the study*

Statistical analysis was performed using R software. Descriptive summary statistics were calculated, and paired t‐tests were conducted for baseline PHQ‐9 and GAD‐7 scores, compared to final scores for all patients who had two or more assessments completed. A *p* value of < 0.05 was deemed to be statistically significant.

## 3 Results

### 3.1 Reach

During the 1‐year period, 148 patients enrolled in the CoCM program and 73 patients graduated. The remainder of patients were still enrolled. Of the initial 148 patients, the majority were female (*n* = 111, 75%) and Black/African American (*n* = 92, 62%) (Table 2). The mean age was 48 years (SD, 15; range 18−78) with a predominance of 50−64 year olds (*n* = 54, 37%) and 36−49 year olds (*n* = 43, 29%). Most patients had private insurance (*n* = 119, 80%). Table 2 provides additional context on the demographic characteristics of individuals reached by the CoCM program.

**Table 2:** Patient demographics, insurance status, and baseline scores. (*N* = 148).

		*N* (%)
Sex		
	Female	101 (75.0%)
	Male	47 (25.0%)
Race		
	Black	92 (62.2%)
	White	31 (20.9%)
	Other	17 (11.5%)
	Declined	8 (5.4%)
Insurance		
	Dual Medicare/Medicaid	9 (6.1%)
	Medicare	20 (13.5%)
	Private	119 (80.4%)
Baseline PHQ‐9		
	0−4 (Minimal)	7 (4.7%)
	5−9 (Mild)	26 (17.6%)
	10−14 (Moderate)	43 (29.1%)
	15−19 (Moderately severe)	42 (28.3%)
	20+ (Severe)	26 (17.6%)
Baseline GAD‐7		
	0−4 (Minimal)	3 (2.0%)
	5−9 (Mild)	22 (14.9%)
	10−14 (Moderate)	53 (35.8%)
	15+ (Severe)	66 (44.6%)
Baseline PHQ and GAD ≥ 10		95 (64.2%)

### 3.2 Effectiveness

All patients had PHQ‐9 and GAD‐7 scores at the time of referral. About three‐quarters of patients had a PHQ‐9 score ≥ 10 (*n* = 111, 75%), and 80% of patients had a GAD‐7 score ≥ 10 (*n* = 119) (Table 3). About 64% (*n* = 95) had both PHQ‐9 and GAD‐7 scores ≥ 10. The mean PHQ‐9 for patients with depression was 16.1 (SD, 4.1), and the mean GAD‐7 for patients with anxiety was 15.3 (SD, 3.3).

**Table 3:** Reach, effectiveness, adoption, and implementation of collaborative care program. (*N* = 148).

		*N* (%) or Mean (SD)	*p* Values
Reach			
	Patients enrolled	148	
Effectiveness			
	Mean difference in PHQ‐9 scores, all patients (*N* = 148)	−4.8 (5.8)	< 0.001
	Mean difference in GAD‐7 scores, all patients (*N* = 148)	−5.6 (5.7)	< 0.001
	Mean difference in PHQ‐9 scores, graduated patients (*N* = 73)	−7.3 (5.4)	< 0.001
	Mean difference in GAD‐7 scores, graduated patients (*N* = 73)	−7.9 (6.0)	< 0.001
	Mean difference in PHQ‐9 scores, patients w/initial score > 10 (*N* = 111)	−6.0 (5.6)	< 0.001
	Mean difference in GAD‐7 scores, patients w/initial score > 10 (*N* = 119)	−6.1 (5.8)	< 0.001
Adoption			
	Attendings who referred to CoCM	20/40 (50%)	
	Residents who referred to CoCM	37/117 (32%)	
	Clinicians who referred to CoCM	57/157 (36%)	
Implementation			
	Median number of days enrolled	57 (IQR = 70)	
	Mean number of encounters per month	2.4 (1.7)	
	Mean number of assessments per month	1.4 (1.1)	
	Proportion of patients with > 1 assessment	75/148 (51%)	
	Patients referred to long‐term therapy	114 (77%)	
	Graduated, referred patients that started long‐term therapy	26/54 (48.1%)	
	Patients discussed per case review	2.3 (1.4)	
	Number of case reviews per enrolled patient	0.60 (0.93)	

Half (51%) of patients had at least one follow‐up PHQ‐9 score and 51% at least one follow‐up GAD‐7 score. Overall, PHQ‐9 (mean −4.8, SD 5.8, *p* < 0.001) and GAD‐7 (mean −5.6, SD 5.7, *p* < 0.001) scores decreased. For the subgroup of patients with scores ≥ 10 at baseline, scores also improved (PHQ‐9: mean −6.0, SD 5.6, *p* < 0.001; GAD‐7: mean −6.1, SD 5.8, *p* < 0.001). Patients who graduated from the program had slightly larger decreases in PHQ‐9 (mean −7.3, SD 5.4, *p* < 0.001) and GAD‐7 (mean −7.9, SD 6.0, *p* < 0.001) scores. Of the graduated patients, 35 patients graduated due to symptomatic improvement, connection to long‐term care, or both.

### 3.3 Adoption

36% (57/157) of PCCs referred patients to the program. Adoption was higher among attending physicians and advanced practice nurses (50%; 20/40) than resident physicians (32%; (37/117).

### 3.4 Implementation

Social workers had a mean of 3.7 clinical encounters (SD, 2.9) per patient, or about 2.4 (SD, 1.7) encounters per month. The PHQ‐9 and GAD‐7 were administered 1.4 times (SD, 1.1) per month. For three‐quarters of patients (*n* = 114), social workers recommended long‐term therapy. 34% of these patients attended a long‐term therapy session within the study period. Among graduated patients, more patients (48%) attended a long‐term therapy session. For patients that graduated, the median length of time in the program was 57 days (IQR = 70 days).

During the study period, 41% (*n* = 61) of patient cases were reviewed with the psychiatrist. There were 89 case review sessions and some patients were discussed multiple times (range, 2−6). On average, 2.3 patients were discussed at each case review. The psychiatrist provided medication recommendations in 89% of discussions.

## 4 Discussion

A team of researchers evaluated the first year implementation of CoCM for adults with depression and/or anxiety in an urban academic general internal medicine clinic with a majority of minority patient population. The team's results demonstrated good adherence to the provision of measurement‐based care and a reduction in depression and anxiety symptoms for all patients that enrolled in the CoCM program, and a greater reduction for patients that graduated from the program. We also identified areas for improvement, such as adoption rates and increasing the number of case reviews conducted.

During the study period, the CoCM program reached about 12 patients with newly diagnosed moderate‐severe depression and/or anxiety per month. The demographics of the patient population showed that the program reached a diverse group of patients in terms of age and race/ethnicity. Consistent with other studies, the patients were predominantly female [7, 12]. The importance of this program's reach is magnified by the fact that the clinic is located in a mental health professional shortage area [13]. Compared to the overall clinic patient population, the CoCM program enrolled a higher proportion of female patients, possibly due to a higher incidence of depression and anxiety in women. In addition, the CoCM program enrolled a higher percentage of black patients than the primary care clinic, possibly indicating more barriers to mental health care access for that population.

The team's results add more support for the expansion of CoCM programs in primary care and in academic primary care clinics. This CoCM program was effective in improving depression and anxiety symptoms at an urban academic teaching clinic that serves a majority minority patient population. Previously, few real‐world studies have examined the effectiveness of CoCM for depression and have had mixed findings. A study of eight primary care clinics at an academic medical center, which implemented a CoCM for any mental health concern, found remission rates of 32% for patients with depression after 12 months and 39% for patients with anxiety [14]. However, a study in the Veteran's Affairs system reported no significant difference in remission rates at 9 months [15]. In this study, it is important that improvement in PHQ‐9 and GAD‐7 scores occurred in all patients enrolled in CoCM, and not just the graduated population, since many patients may not complete CoCM.

Other studies have identified barriers to adoption of CoCM programs, such as increased workload and provider concerns about psychotherapy [16]. Early clinical improvements and investing in full‐time staff were shown to be crucial for adoption [17]. Another study found that CoCM adoption is often stymied by a lack of awareness regarding billing codes that would finance programs [18]. On the clinicians' side, the team's data showed that over one‐third of physicians referred to the CoCM program after 12 months, with higher rates among attending clinicians, closer to rates seen at the VA in its mandatory implementation of CoCM [19]. Resident physicians were less likely to refer patients to CoCM, which may be related to a lack of awareness. Adoption could be increased by improving communications to the resident physicians about CoCM.

Overall, implementation occurred with good fidelity. Results showed that social workers were able to meet with patients at a higher‐than‐expected frequency of 2.4 times per month (vs. 2 times per month). In addition, patients were assessed via the PHQ‐9 and GAD‐7 1.3 times per month, which again exceeded the expected frequency of once per month. These findings are comparable to other CoCM programs in the literature [14, 15]. The team's results showed a limitation in implementation, which was the lack of case reviews for all enrolled patients. Potential explanations include time limitations and the knowledgeable discretion of the experienced CoCM social workers.

Although this study contributes to the understanding of CoCM, limitations exist. The timeframe of the study limited its ability to assess program maintenance, including potential loss to follow‐up, and metrics such as patient enrollment and provider engagement could have been trended over time. The study was retrospective, and the data were extracted from documentation of encounters between providers and patients. There was variation between providers in how the encounters were documented. This limitation was mitigated by the use of standardized forms. In addition, we did not collect data on depression and anxiety remission as assessed by a clinician. The study was conducted at a single academic center in an urban setting, and therefore, the results might not be generalizable.

Overall, this study reinforces the effectiveness of the CoCM in an urban academic teaching clinic with a majority minority population as a method of delivering integrated behavioral health care and details metrics used to assess reach, adoption, and fidelity to the CoCM. Ongoing evaluation will be needed as staffing and patient volumes fluctuate. Future work should also assess maintenance, including program sustainability and long‐term patient outcomes.

## Conflicts of Interest

The authors declare no conflicts of interest.

## Data Availability

The data that support the findings of this study are available on request from the corresponding author. The data are not publicly available due to privacy or ethical restrictions.
